# Supplementary material for: Cooperative colloidal self-assembly of metal-protein superlattice wires
Source: Nat Commun. 2017 Sep 22;8:671. doi: 10.1038/s41467-017-00697-z (PMC5610313; doi:10.1038/s41467-017-00697-z)
Supplement: Supplementary file 1 — Supplementary Information [file 41467_2017_697_MOESM1_ESM.pdf]

Supplementary Figures

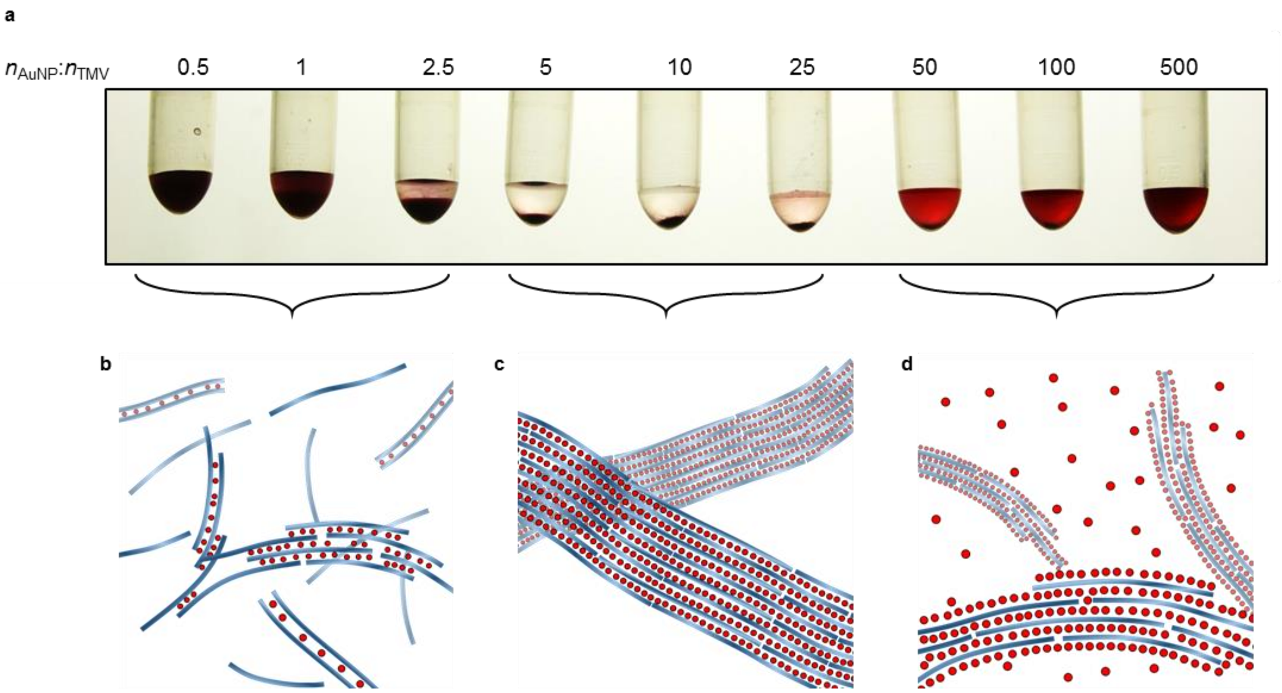

Supplementary Figure 1 | Dialyzed samples. a, AuNP-TMV samples collected into Eppendorf tubes after dialysis. The nanoparticle stoichiometric ratio is indicated in the figure. The total amount of AuNP is the same in every sample. The samples can roughly be divided into three categories: **b**, Significant excess of TMV. The samples include free TMVs and incomplete superlattice structures. **c**, Appropriate stoichiometric ratio for forming complete superlattices. Neither free TMVs or AuNPs are present in significant amounts. **d**, Significant excess of AuNP. The free AuNP gives the supernatant a ruby red colour.

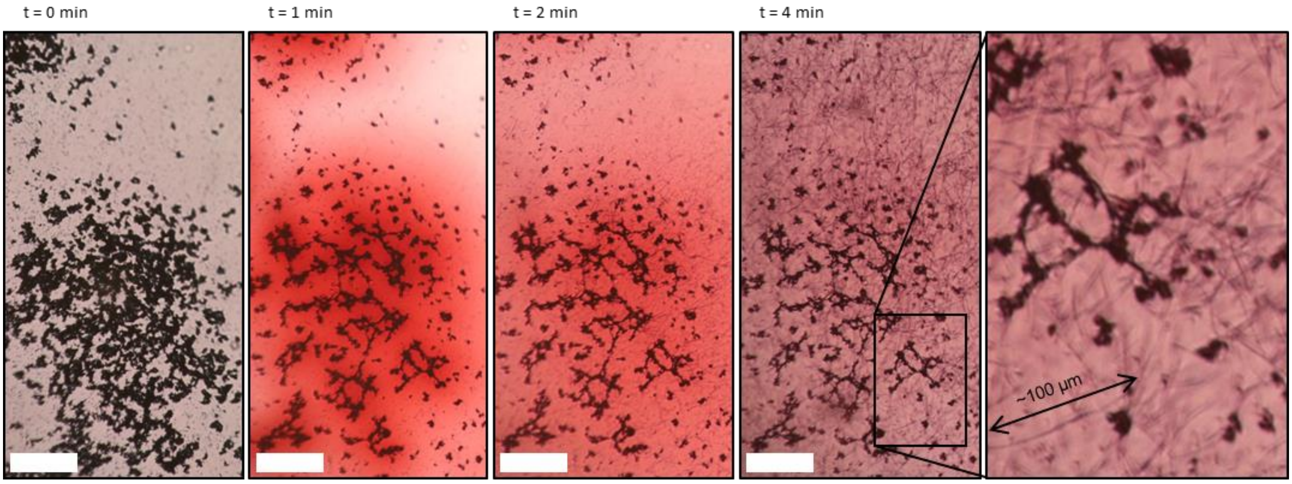

Supplementary Figure 2 | *In-situ* time lapse of AuNP-TMV mixture in dialysis. The initial NaCl concentration in the sample is 500 mM (t = 0) and the dialyte NaCl concentration is 0 mM. The time lapse reveals that AuNPs are first released from the aggregates (red colour at t = 1 min) and then forming superlattices together with the dispersed TMV (gradual colour change and observed fibrillary structures at t = 4 min). Scale bars are 100 µm.

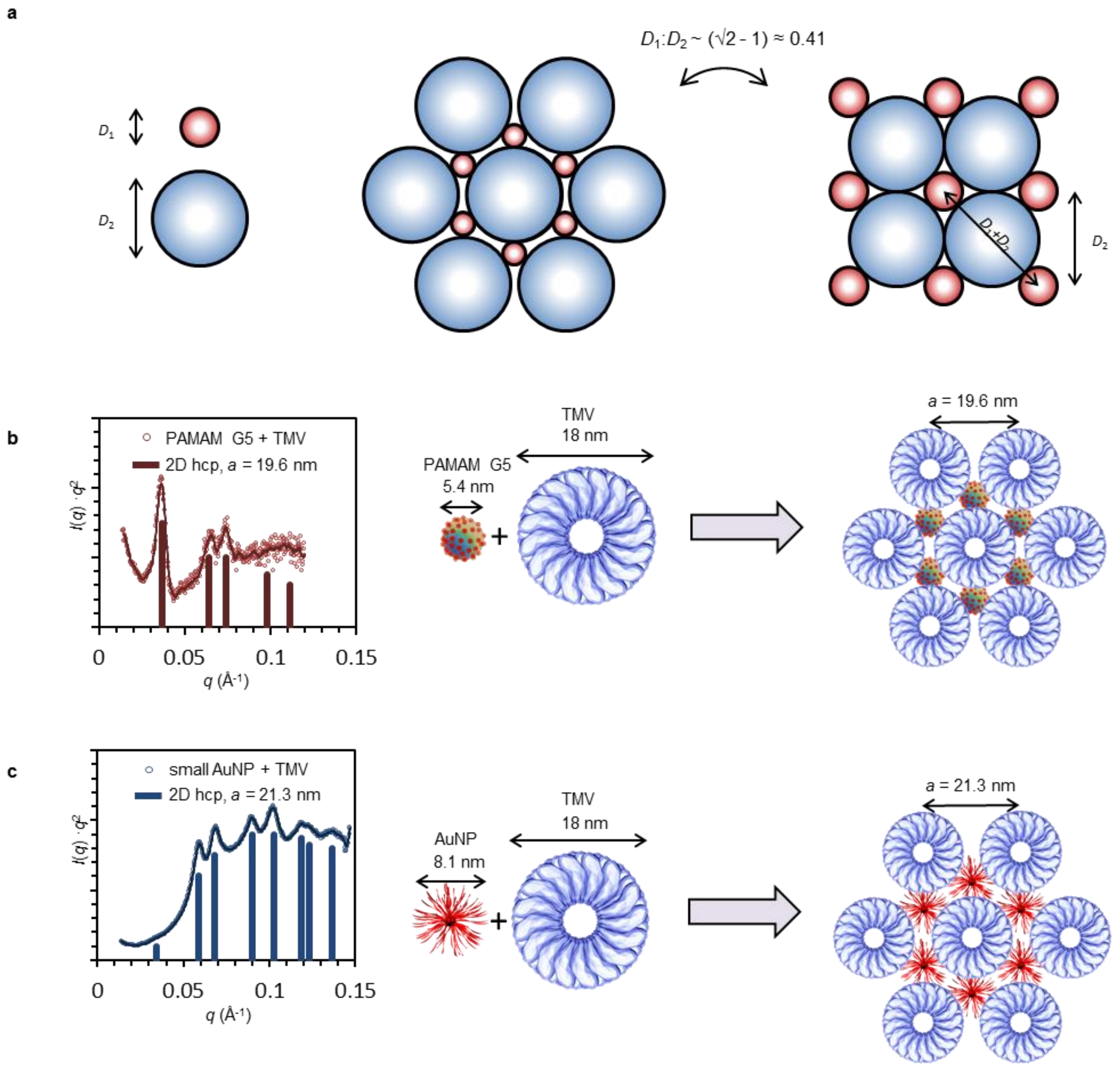

Supplementary Figure 3 | Hexagonal TMV lattices are formed when smaller cationic particles are used. a, The particle size ratio affects the close packed structure. A close packed square lattice can be obtained when the spherical particle is large enough to efficiently interact with four rod-like TMV particles. **b**, SAXS data showing that poly(amidoamine) generation 5 dendrimers (PAMAM G5) form a hexagonal lattice together with TMV. **c**, SAXS data showing that small AuNPs (D_{core} = 2.6 nm, D_{H} = 8.1 nm) form a hexagonal lattice together with TMV. The lattice constant *a* is smaller than would be assumed from the particle sizes, which is explained by compaction of the soft PAMAM dendrimer (**b**) and compaction of the flexible ligands of the AuNP (**c**) between the rigid TMVs.

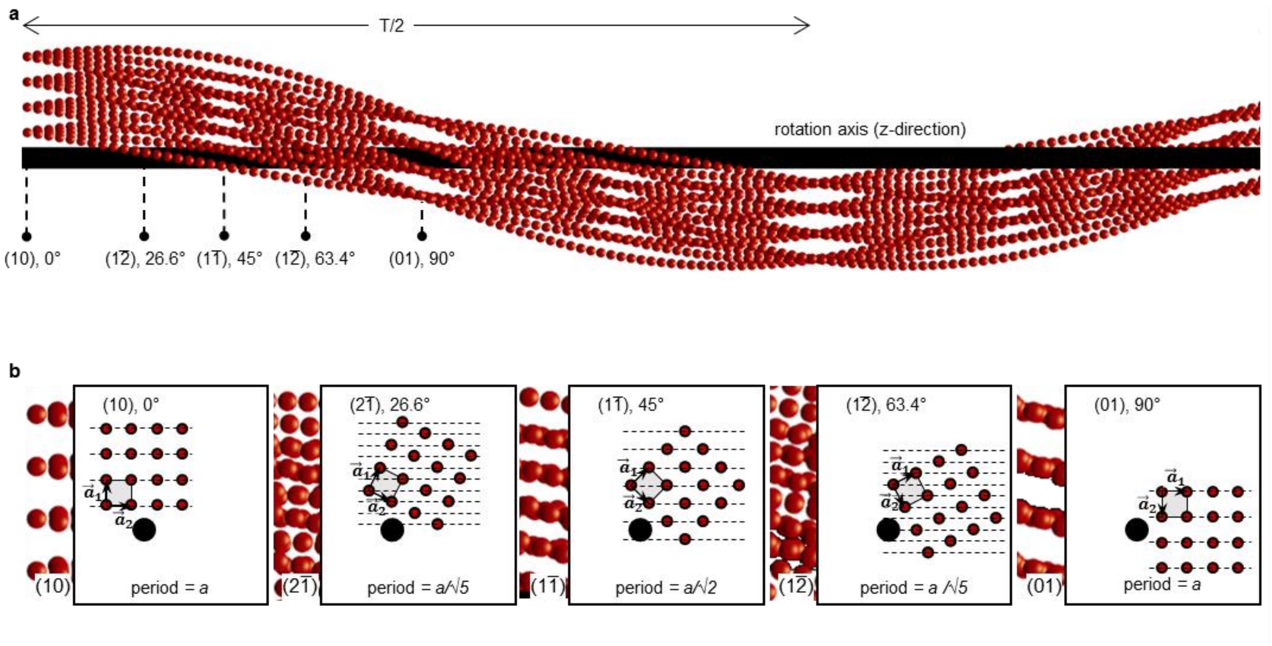

Supplementary Figure 4 | Lattice periods that can be identified from the projection of the twisted 2D superlattice. The pitch T can be estimated from the distance between the observed lattice planes, which are identified by the lattice plane distance (period). **a**, A helically twisted finite 2D square lattice. The cross sectional size of the model lattice is 4×4 nanoparticles. **b**, Close up images from the indicated positions in **a** and corresponding cross sections from the structure viewed along the rotation axis direction. The dashed lines indicate the lattice planes seen at the indicated positions in **a**.

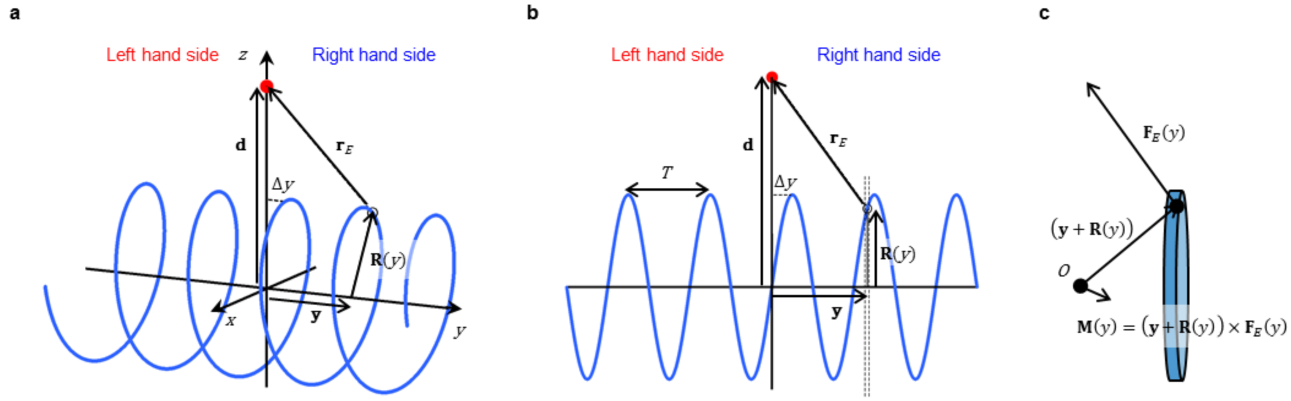

Supplementary Figure 5 | Schema of a helical line charge and an opposite point charge. a and **b**, The charge configuration viewed from different directions. The interaction is inspected for every segment (**b**) of the helix. **c**, The electrostatic forces acting on a segment causes a bending moment $\mathbf{M}(y)$ about the origo O.

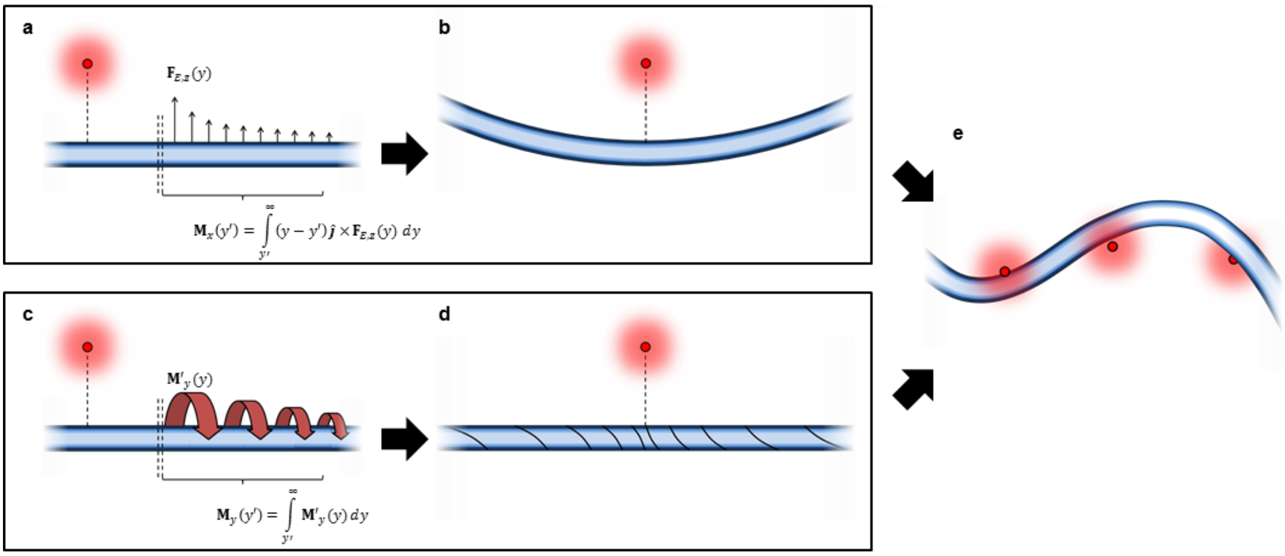

Supplementary Figure 6 | Simultaneous bending and torsion yields helicity. a, The *z* components of the attractive forces form the bending moment $\mathbf{M}'_x(y)$ acting on the segment at y. **b**, The point charge strives to bend a rod with an opposite helical charge distribution. **c**, The torques $\mathbf{M}_y(y')$ about the *y* axis form the bending moment $\mathbf{M}'_y(y)$ acting on the segment at y. text. **d**, The point charge strives to twist a rod with an opposite helical charge distribution. Torsion alone does not cause a deflection of the rod. **e**, A combination of torsion and bending leads to helical deformation.

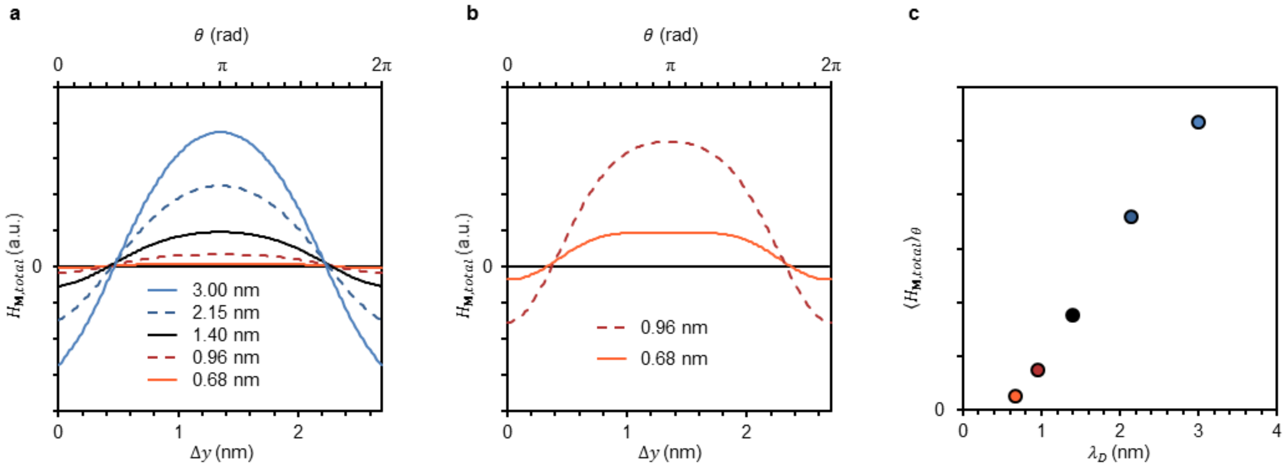

Supplementary Figure 7 | Helicity of the attractive forces acting on a helical charge distribution. a, The total helicity of the forces acting on rod with a helical charge distribution is depending on the position Δy of the opposite point charge relative to the helix. The Δy average of the helicity $\langle H_{\mathbf{M},total}\rangle_\theta$ is positive, referring to an average right handed twist. The λ_D values used in the calculations are indicated in the legend. **b**, Close-up of the values corresponding to small λ_D values. **c**, The helicity of the average bending moment is magnified at lower ionic strengths (corresponding to high λ_D) due to stronger electrostatic interaction.

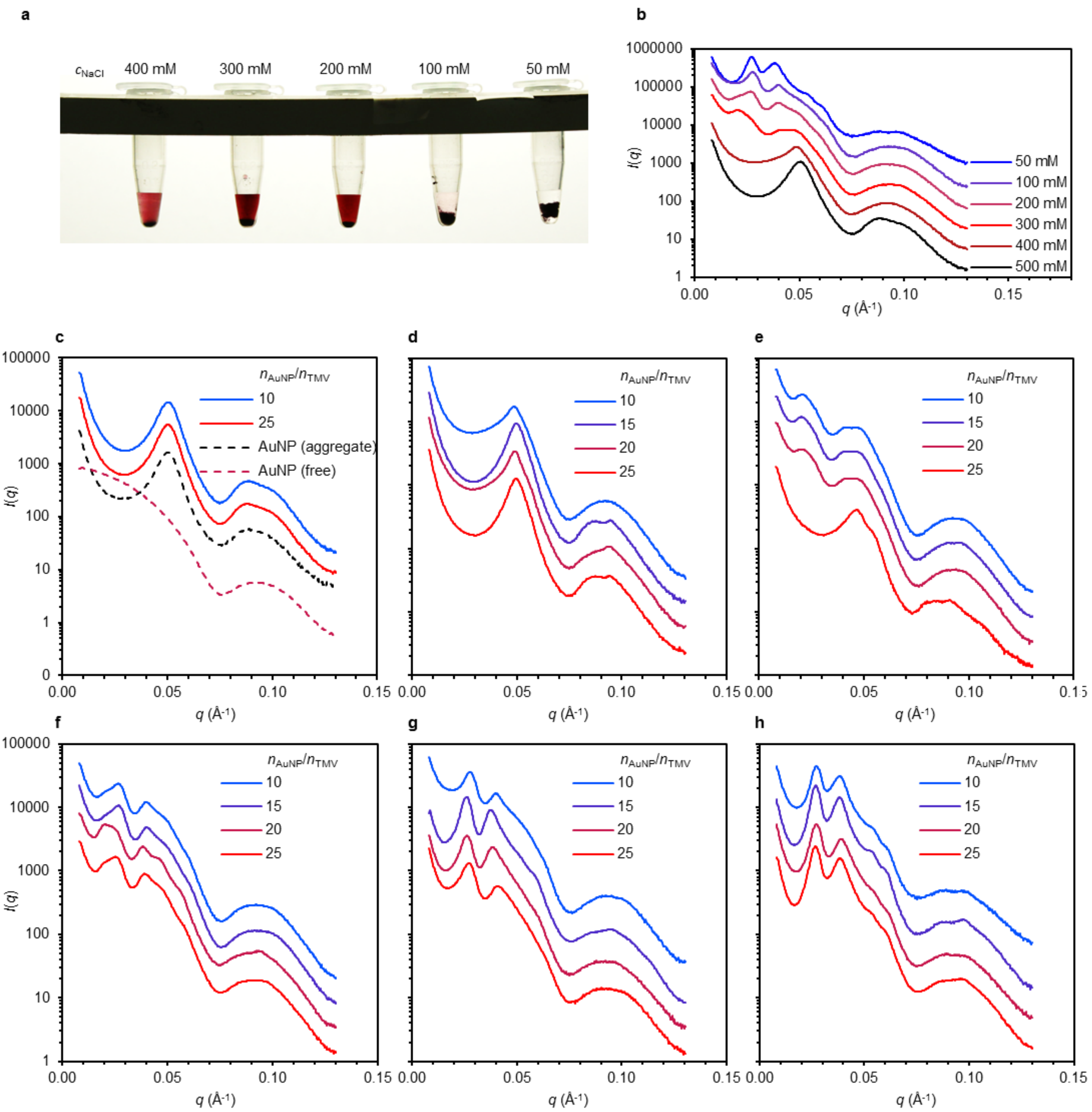

Supplementary Figure 8 | Continuous formation of superlattice structures. a, AuNP-TMV samples (n_{AuNP}/n_{TMV} ~ 20) dialyzed sequentially to different NaCl concentrations. The amount of AuNPs and TMV is the same in every sample. Samples containing dispersed AuNPs have a red supernatant. At 400 mM some of the AuNPs are still aggregated on the bottom of the tube. The TMVs form sedimenting assemblies at c_{NaCl} ~ 300 mM, but do not bind all of the AuNPs. All dispersed AuNPs are bound from the supernatant at c_{NaCl} ~ 50 mM. **b**, The SAXS characteristics of the self-assembly at different ionic strengths (n_{AuNP}/n_{TMV} ~ 10). At $c_{NaCl} \geq 400$ mM no significant lattice structure can be identified. At $c_{NaCl} \leq 100$ mM the SAXS data matches unambiguously with the 2D square lattice. At c_{NaCl} ~ 200–300 mM a peak corresponding to d ~ 30 nm is present while it disappears at $c_{NaCl} < 50$ mM. **c-h**, The SAXS profiles at different c_{NaCl} for the sample series with n_{AuNP}/n_{TMV} 10–25 (indicated in the legend). The differences in the SAXS profiles (and hence the nanostructures) is more sensitive to the c_{NaCl} than to the n_{AuNP}/n_{TMV}. Some evaporation of water might occur from the samples during the measurement regardless of careful sealing. Therefore the nominal c_{NaCl} might be an underestimation of the actual c_{NaCl}. The dispersed AuNPs (“AuNP free” in (**c**)) are measured at a low ionic strength.

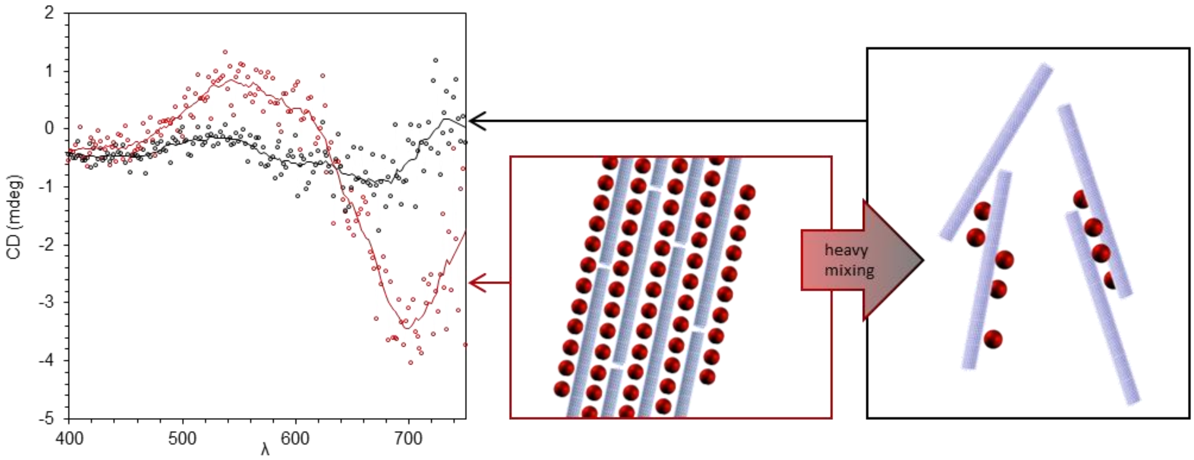

Supplementary Figure 9 | CD signal is washed out by shear. A change in the CD spectrum was observed when superstructures were broken by pumping with a pipette.

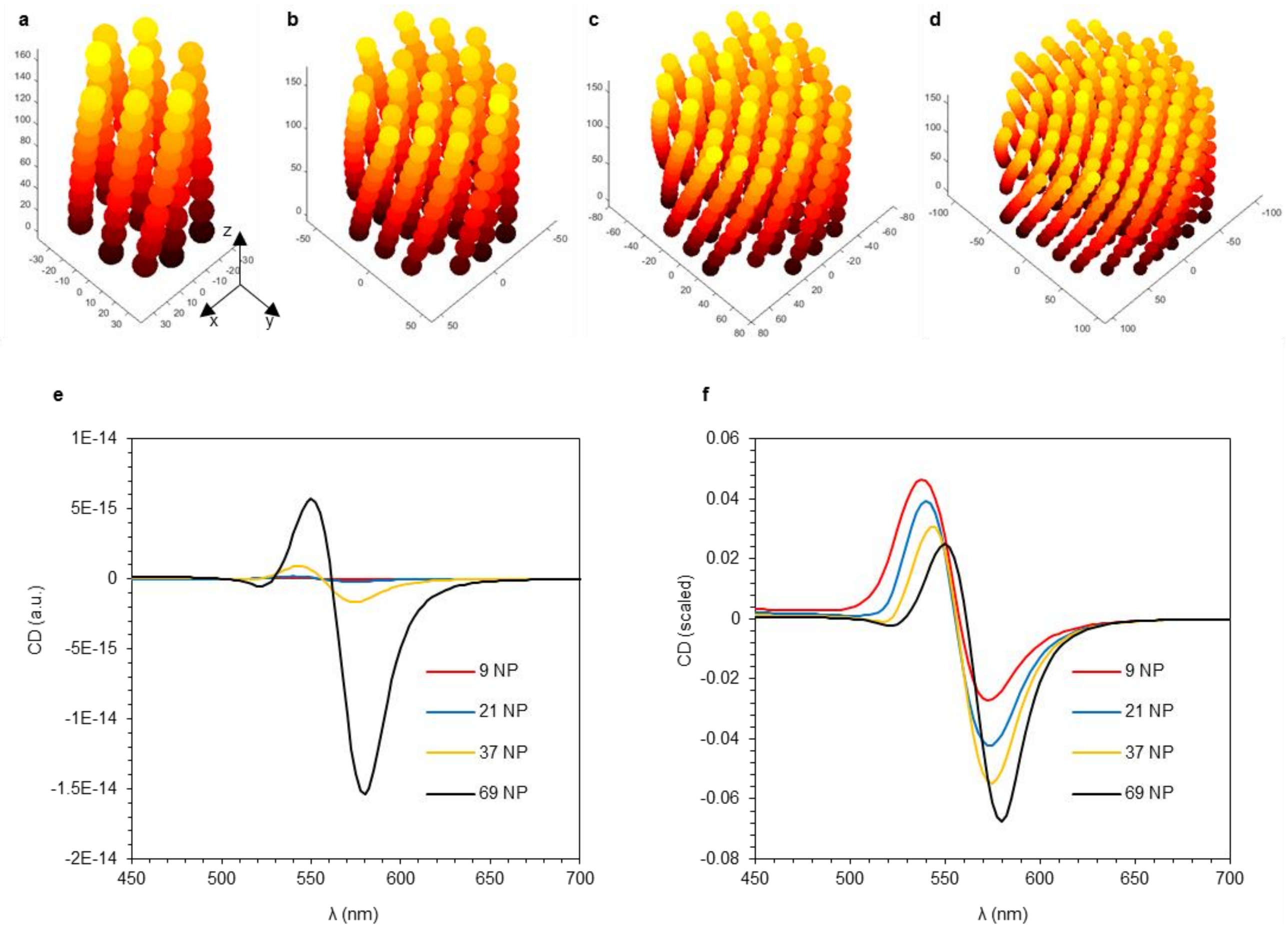

Supplementary Figure 10 | Modelling the superlattice circular dichroism. Generated right handed finite superlattice models with ω = 0.25 ° nm^{-1} and ten layers of nanoparticles in *z*-direction (rotation axis). The simulations were done for models with four different cross sections (bases), *i.e.* the modelled AuNP assemblies had different widths. All the models have a cross sectional square lattice with a = 23.13 nm and an interparticle distance of 16 ± 1.6 nm for the AuNPs in the individual rows in the rotation axis direction. The number of AuNPs in the superlattice base are 9 in **a**, 21 in **b**, 37 in **c**, and 69 in **d**. **e**, Simulated CD spectra of all the models. **f**, Scaled simulated CD spectra. The spectra are scaled by the dividing with the average magnitude of the CD spectra to highlight the difference in the shape of the spectra. The simulation shows that for the probed ω values the characteristic peak-dip feature remains independent of ω. Both the position and the magnitude of the observed features in the CD spectra vary as a function of the superlattice size. The experimental spectra in Fig. 3 show broad features, which can be explained by the observed distribution of superlattice sizes.

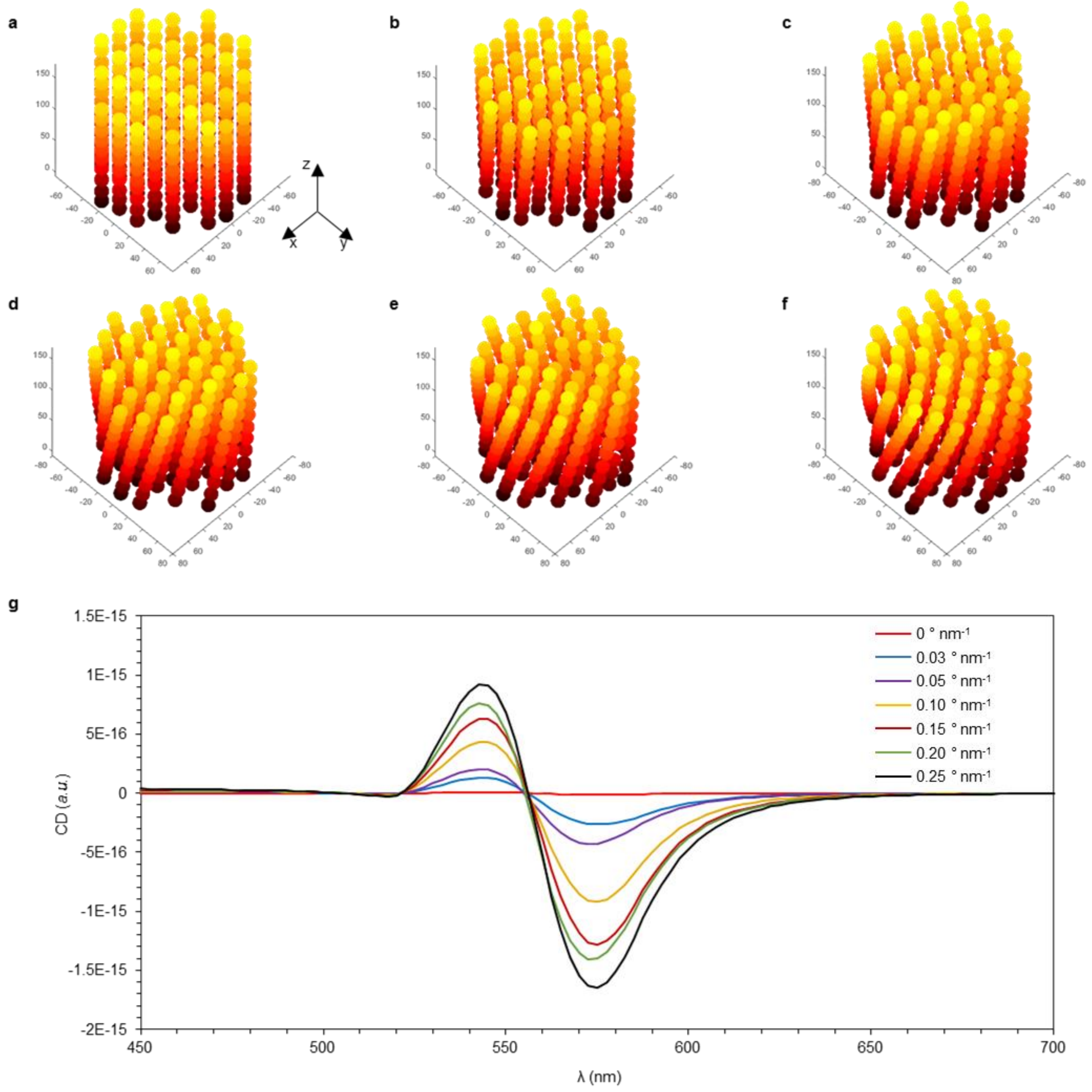

Supplementary Figure 11 | Modelling the superlattice circular dichroism. a-f, Generated right handed finite superlattice models with ω = [0 ° nm^{-1}, 0.05 ° nm^{-1}, 0.10 ° nm^{-1}, 0.15 ° nm^{-1}, 0.20 ° nm^{-1}, 0.25 ° nm^{-1}] and ten layers of nanoparticles in *z* direction (rotation axis). All the models include 370 AuNPs and have a cross sectional square lattice with *a* = 23.13 nm and an interparticle distance of 16 ± 1.6 nm for the AuNPs in the individual rows. **g**, Simulated CD spectra of all the models. The simulation show that only the magnitude of the peak-dip feature is sensitive to a variation of ω. Peak position or width does not change as a function of ω.

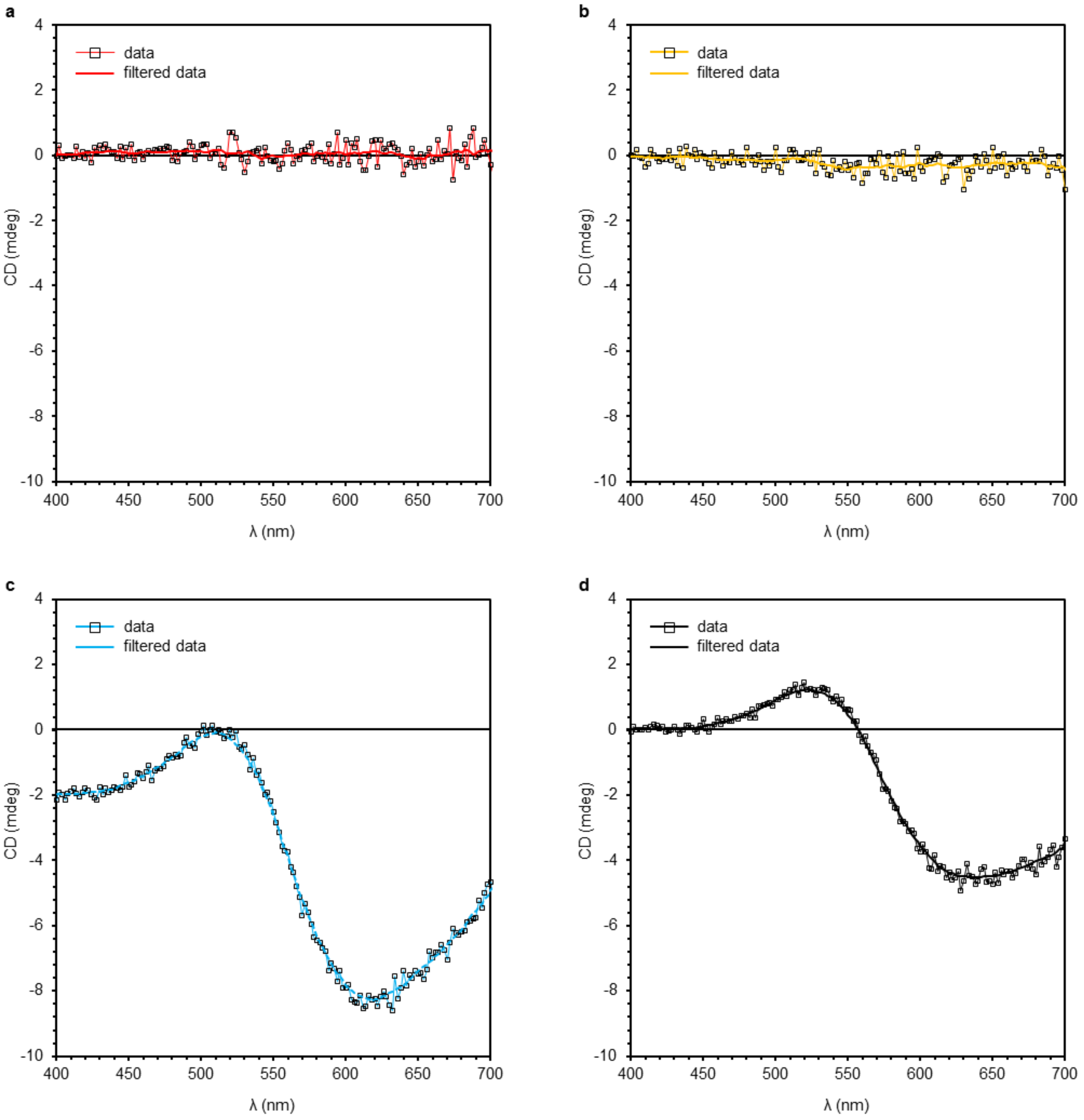

Supplementary Figure 12 | Original data and Savitzky–Golay filtered data. a, Dissolved AuNP in aqueous solution. **b,** AuNP-TMV sample at 400 mM sodium chloride solution buffered with 10 mM sodium acetate. **c,** AuNP-TMV sample at 200 mM sodium chloride solution buffered with 10 mM sodium acetate. **d,** AuNP-TMV sample at 0 mM sodium chloride solution buffered with 10 mM sodium acetate. $n_{\mathrm{AuNP}}/n_{\mathrm{TMV}}$ is ~ 25 in all samples.

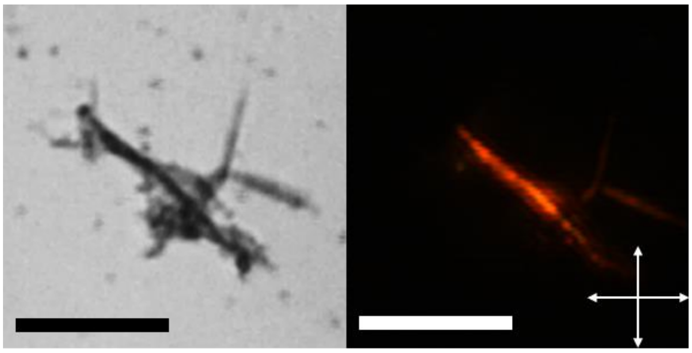

Supplementary Figure 13 | Kinetically locked aggregates of magnetic superlattice wires. Due to the unoptimised Fe_3O_4 nanoparticle functionalization some superlattice wires are kinetically locked in aggregates. Such superlattice wires are not able to align in a magnetic field. Bright field optical microscope image (left) and polarized optical microscope image (right). The direction of the crossed polarizers are indicated by double arrows. Scale bars: 50 µm.

Supplementary Note

Bending and torsional forces exerted on a helical line charge by a point charge in a static configuration.

To get qualitative insight of the forces exerted on a TMV due to the close vicinity of a AuNP, let us simplify the situation to include only a rigid rod with a negative helical charge distribution and an opposite point charge. We approach the problem by assuming a static configuration. Physically this would mean that all forces and torques are counteracted by reaction forces, which allows us to inspect the configuration without the complications included in elastic deformation or relative motion of the configuration. The static approach is also motivated by the observation that TMV, which corresponds to the rod with a negative helical charge distribution, is relatively rigid and undergoes only a minor deformation when incorporated to the helical assemblies.

The point charge is located on the *z* axis at a constant distance **d** from the origo *O*. Let us define an oppositely charged helix having its center at *O* and with a rotation axis defined by the *y* axis. Every point on the helix is now defined by the vector $\mathbf{R}(y)$ that rotates about the *y* axis, *i.e.*

$$\mathbf{R}(y) = R\left[\sin\left(2\pi\frac{y}{T} - \theta\right)\hat{\mathbf{i}} + \cos\left(2\pi\frac{y}{T} - \theta\right)\hat{\mathbf{k}}\right], \quad (1)$$

where T is the helical pitch and $\theta = 2\pi\Delta y/T$ according to Fig. S5.

The distance $\mathbf{r}_E$ from $\mathbf{R}(y)$ to the point charge is

$$\mathbf{r}_E(y) = \mathbf{d} - \mathbf{y} - \mathbf{R}(y) = d\hat{\mathbf{k}} - y\hat{\mathbf{j}} - R\sin\left(2\pi\frac{y}{T} - \theta\right)\hat{\mathbf{i}} - R\cos\left(2\pi\frac{y}{T} - \theta\right)\hat{\mathbf{k}}. \quad (2)$$

The electrostatic force exerted on the helix by the point charge is

$$\mathbf{F}_E(y) = F_E(y)\,\frac{\mathbf{r}_E(y)}{|\mathbf{r}_E(y)|} = A(y)F_E(y)\mathbf{r}_E. \quad (3)$$

$$Ar_E \equiv \hat{\mathbf{r}}_E. \quad (4)$$

$$\begin{aligned} A &= \left[R^2\sin^2\left(2\pi\frac{y}{T} - \theta\right) + y^2 + d^2 - 2dR\cos\left(2\pi\frac{y}{T} - \theta\right) + R^2\cos^2\left(2\pi\frac{y}{T} - \theta\right)\right]^{-\frac{1}{2}} \\ &= \left[R^2 + y^2 + d^2 - 2dR\cos\left(2\pi\frac{y}{T} - \theta\right)\right]^{-\frac{1}{2}} \end{aligned} \quad (5)$$

In order to inspect the torsional forces exerted on the helix we may divide the helix into infinitesimal segments and inspect the net torsional forces on the right hand side and the left hand side of the point charge (Fig. S5b, S5c). Because the charge carried by a single segment is unevenly distributed, each segment causes a bending about O due to the attraction of the point charge. The bending moment $\mathbf{M}'(y)$ about the central point is

$$\mathbf{M}'(y) = \left(\mathbf{y} + \mathbf{R}(y)\right) \times \mathbf{F}_E(y)$$

$$= A(y)F_E(y) \begin{vmatrix} \hat{\mathbf{i}} & \hat{\mathbf{j}} & \hat{\mathbf{k}} \\ R\sin\left(2\pi\frac{y}{T} - \theta\right) & y & R\cos\left(2\pi\frac{y}{T} - \theta\right) \\ -R\sin\left(2\pi\frac{y}{T} - \theta\right) & -y & d - R\sin\left(2\pi\frac{y}{T} - \theta\right) \end{vmatrix} \tag{6}$$

$$= A(y)F_E(y)\left[yd\hat{\mathbf{i}} - Rd\sin\left(2\pi\frac{y}{T} - \theta\right)\hat{\mathbf{j}}\right].$$

We can observe that due to the point charge, any segment of the helix is subject to a bending moment

$$\mathbf{M}'_x(y) = A(y)F_E(y)yd\,\hat{\mathbf{i}} \tag{7}$$

about the *x* axis and a torque

$$\mathbf{M}'_y(y) = -A(y)F_E(y)dR\sin\left(2\pi\frac{y}{T} - \theta\right)\hat{\mathbf{j}} \tag{8}$$

about the y axis. The bending moment is positive for all positive y values and negative for all negative y values. The net bending moment on the right hand side is therefore positive and the net bending moment on the left hand side is negative, implying that the helix strives to bend around the point charge, which is most intuitive, as even a smoothly charged rod would experience a bending around an opposite point charge. Even though this result alone is enough to explain the formation of helical superstructures (a helical arrangement should result from forming zipper-like assemblies including bent rods) the resulting population of superlattices would be a racemic mixture without a preferred helicity. However, we can identify that $\mathbf{M}'(y)$ includes a component $\mathbf{M}'_y(y)$ of torque about the y axis (rotation axis of the helix). $\mathbf{M}'_y(y)$ is a periodic function, and requires therefore a more detailed inspection in order to yield any qualitative information about the net torque on the right hand side and left hand side respectively.

It is important to recognize that a rod can be deformed into a helix by applying simultaneous bending and torsion. The handedness of the torsion defines the handedness of the formed helix (right handed torsion yields a right handed helix, Fig. S6). Therefore we estimate the net effect of the bending moments exerted on the helix by inspecting the helicity $H_{\mathbf{M}}(y)$ of the forces. The convention is that positive helicity denotes a right handed structure, and thus a positive helicity of forces denotes forces that drives a right handed deformation.

We start by inspecting the effect of all torsional and bending forces acting on a segment at y, $y \geq 0$. (The same arguments can similarly be applied to the case $y \leq 0$.) As we are considering a static configuration, we are allowed to make the assumption that the total of all forces and torques exerted on any segment equals to zero. Physically this implies that any force is counteracted by a reaction force. The abovementioned $\mathbf{M}'$ variables result from the attractive force acting on an infinitesimal segment by the point charge. It needs to

be pointed out that $\mathbf{M}'(y)$ is infinitesimally small, and becomes significant only when integrated over an interval.

The significant bending and torque that acts on a segment is that which is transmitted from other parts of the helix (Fig. S6). Therefore, to avoid misinterpretation we now define the **M** variables to describe the (by internal stress) transmitted bending and torque acting on a segment. The bending moments about *x* and *z* axes acting on a segment at y' on the *y* axis are

$$\mathbf{M}_x(y') = M_x(y')\hat{\mathbf{i}} = \int_{y'}^{\infty} (y - y')\,\hat{\mathbf{j}} \times \mathbf{F}_{E,z}(y)dy, \tag{9}$$

and

$$\mathbf{M}_z(y') = M_z(y')\hat{\mathbf{k}} = \int_{y'}^{\infty} (y - y')\,\hat{\mathbf{j}} \times \mathbf{F}_{E,x}(y)dy, \tag{10}$$

and the torque about the *y* axis acting on the segment at y_0 is

$$\mathbf{M}_y(y') = M_y(y')\hat{\mathbf{j}} = \int_{y'}^{\infty} \mathbf{M}'_y(y)\,dy. \tag{11}$$

$\mathbf{F}_{E,z}(y)$ and $\mathbf{F}_{E,x}(y)$ are the *z* and *x* components of $\mathbf{F}_E(y)$. $\mathbf{F}_{E,z}(y)$ is periodically alternating between positive and negative values and $\mathbf{M}_z(y_0)$ is hence small in comparison to $\mathbf{M}_x(y_0)$ and is thus not taken into consideration.

The used definition of helicity $H_{\mathbf{M}}(y_0)$ in this configuration is ambiguous and requires careful treatment. The definition should yield a positive value for net internal forces striving to bend the structure in a right handed manner. Here we choose to define $H(y_0)$ as

$$H_{\mathbf{M}}(y) = M_x(y)M_y(y), \tag{12}$$

in line with the abovementioned identification that helicity here arises from simultaneous bending ($M_x(y)$) and torsion ($M_y(y)$). The connection between $H_{\mathbf{M}}$ and the formation of a helical structure can be understood in terms of deflection from the initial main axis (*y* axis). Axial torsion alone does not cause a round rod to deflect from its initial main axis. Bending does cause deflection from the initial main axis, but does not include any component of helical torsion. If bending and right handed torsion are applied simultaneously, the structure strives to deform in a helical manner. Furthermore, for small deflections (in the elastic regime) the

deflection caused by local bending is directly proportional to $M_x(y)$ as the deflection caused by local torsion is directly proportional to $M_y(y)$, yielding helicity as the *product* of these two.

We can now calculate the total helicity $H_{\mathbf{M},\text{total}}$ as

$$H_{\mathbf{M},\text{total}} = \int_{-\infty}^{\infty} H(y)dy. \tag{13}$$

We know from experiments that in the experimental system consisting of TMVs and AuNPs, the AuNPs are allowed to move with respect to the TMV. Therefore it is meaningful in this consideration to evaluate the θ average helicity $\langle H_{\mathbf{M},\text{total}}\rangle_\theta$, which can be evaluated as

$$\langle H_{\mathbf{M},\text{total}}\rangle_\theta = \int_{0}^{2\pi} H_{\text{total}}\, d\theta. \tag{14}$$

For this we use the screened Coulomb potential for a point charge

$$V_E(r) = \frac{Cq_1}{r}\, e^{-r/\lambda_D}, \tag{15}$$

where λ_D is the Debye length

$$\lambda_\text{D} = \left(\varepsilon kT/e^2 \sum_i c_i z_i^2\right)^{1/2}, \tag{16}$$

which gives an electric field

$$E(r) = \frac{Cq_1}{r}\left(\frac{1}{\lambda_\text{D}} + \frac{1}{r}\right) e^{-r/\lambda_\text{D}}, \tag{17}$$

and an electrostatic force

$$F_E = \frac{Cq_1q_2}{r}\left(\frac{1}{\lambda_\text{D}} + \frac{1}{r}\right) e^{-r/\lambda_\text{D}}. \tag{18}$$

$C = 1/(4\pi\varepsilon_0\varepsilon_\text{r})$, ε_0 is the vacuum permittivity, and ε_r is the relative permittivity for the media. The inspection was done for values λ_D = [0.68 nm, 0.96 nm, 1.4 nm, 3 nm] which correspond to c_{NaCl} = [200 mM, 100 mM, 50 mM, 10 mM]. The radius R was chosen as 8.5 nm and d was chosen as 9.5 nm. The magnitude of the charges in the system were left undefined, hence the results (Fig. S7) are presented in arbitrary units (*a.u.*).

This consideration shows that a point charge can exert both bending and torsional forces on a helical charge distribution. The asymmetric electrostatic interaction is the most probable explanation for the formed helical superstructure, because when torsion and bending are applied on an elastic rod simultaneously, the rod

deforms in a helical manner. The handedness of the applied torsion yields the handedness of the helical deformation (right handed torsion together with bending yields a right handed helix). In this inspection we observe that the helicity is either right handed or left handed, depending on the relative position of the point charge, but the θ-average (= Δy-average) helicity remains positive, corresponding to an average right handed twist. To compare this simplified charge configuration with that of a cationic nanoparticle and TMV is far from trivial. However, our experimental data shows that the strong electrostatic interaction dominates the self-assembly, leading to the conclusion that the right handed helical structures result from the asymmetric electrostatic interaction between a cationic spherical nanoparticle and TMV, which carry a helical charge distribution. As said, merely the symmetric attraction can explain the formation of helical superlattices, but an asymmetric interaction is needed to explain the preferred handedness. The details of the electrostatic potential map of TMV is on sub nanometer level, whereas the observed pitch of the helical superlattices is in micrometer scale. We believe that a complete understanding of the formation of the observed helical superlattice structures would require detailed, but large scale molecular simulations that are beyond the state of the art.
