# Supplementary material for: Cooperative colloidal self-assembly of metal-protein superlattice wires
Source: Nat Commun. 2017 Sep 22;8:671. doi: 10.1038/s41467-017-00697-z (PMC5610313; doi:10.1038/s41467-017-00697-z)
Supplement: Supplementary file 3 — Supplementary Files [file 41467_2017_697_MOESM3_ESM.pdf]

Description of Additional Supplementary Files

File Name: Supplementary Movie 1
Description: Cryogenic transmission electron microscope tilt series of a AuNP-TMV superlattice wire

File Name: Supplementary Movie 2
Description: Cryogenic electron tomographic reconstruction of a AuNP-TMV superlattice wire.
